# Numerical simulation of gonadal vein hemodynamics in nutcracker syndrome-associated varicocele: effects of Valsalva maneuver and clinical implications

**DOI:** 10.3389/fbioe.2026.1828724

**Published:** 2026-05-18

**Authors:** Yun-Hua Ji, Guo-Qiu Liu, Xu-Yan Guo, Hao-Zhong Hou, Wei Zhang, Da-Li He, Tong-Tong Wang, Bo Zhang, Pei-Wen Shi, Zhen Yao

**Affiliations:** 1 Department of Urology, Tangdu Hospital, The Fourth Military Medical University, Xi’an, Shaanxi, China; 2 School of Energy and Power Engineering, Xi’an Jiaotong University, Xi’an, China; 3 Department of Health Management, Tangdu Hospital, The Fourth Military Medical University, Xi’an, Shaanxi, China

**Keywords:** computational fluid dynamics, gonadal vein, hemodynamics, nutcracker syndrome, Valsalva maneuver, varicocele, wall shear stress

## Abstract

**Objectives:**

Nutcracker syndrome (NCS) is a vascular disorder characterized by compression of the left renal vein (LRV), frequently complicated by varicocele in male patients. The Valsalva maneuver is routinely used to provoke venous reflux during ultrasound diagnosis, yet the hemodynamic response of the gonadal vein throughout a complete Valsalva cycle remains poorly characterized. This study aims to quantitatively elucidate the hemodynamic mechanisms by which intra-abdominal pressure elevation—simulated via the Valsalva maneuver—exacerbates gonadal venous reflux in NCS-associated varicocele.

**Methods:**

Fifty-seven patients with NCS and concomitant varicocele were consecutively enrolled. Doppler ultrasonography was performed to measure anatomical and hemodynamic parameters, including aortomesenteric angle, LRV diameter, stenotic diameter, peak velocities, and gonadal vein diameter under resting and Valsalva conditions. A representative three-dimensional computational fluid dynamics (CFD) model incorporating the inferior vena cava, LRV, and gonadal vein was reconstructed from contrast-enhanced CT data. A physiologically relevant time-varying pressure boundary condition was applied at the gonadal vein inlet to simulate a complete Valsalva cycle. Pressure, velocity, wall shear stress, and streamline distributions were systematically analyzed.

**Results:**

Clinical data revealed significant anatomical compression and hemodynamic abnormalities, with gonadal vein diameter increasing from 3.0 ± 0.94 mm at rest to 3.86 ± 1.07 mm during Valsalva. CFD simulations demonstrated that during the Valsalva plateau phase, pressure at the gonadal vein inlet reached 6,000 Pa, with a pressure gradient of approximately 7,600 Pa across the LRV stenosis. Peak WSS at the stenosis increased 12-fold, exhibiting marked spatial heterogeneity. Velocity at the stenosis reached 2.7 m/s, with pronounced flow separation and disturbed streamlines developing downstream, which reduced the effective flow volume by 50%–60%.

**Conclusion:**

The Valsalva maneuver induces a high-pressure-negative-pressure-recompression vicious cycle across the LRV stenosis, with elevated upstream pressure increasing venous congestion risk and downstream negative pressure exacerbating stenotic collapse. The 12-fold WSS elevation far exceeds physiological thresholds, potentially precipitating cumulative endothelial injury. Recirculation zone formation compromises venous return and promotes thrombogenesis. Crucially, these hemodynamic perturbations are not confined to diagnostic maneuvers but are likely mirrored during daily activities that elevate intra-abdominal pressure (e.g., running, jumping), explaining the progressive symptom worsening and activity-related discomfort in NCS patients. These findings provide a hemodynamic rationale for dynamic clinical assessment.

## Introduction

Nutcracker syndrome (NCS), also termed left renal vein entrapment syndrome, is a vascular disorder characterized by mechanical compression of the left renal vein (LRV), resulting in impaired venous outflow and a spectrum of clinical manifestations (Heilijgers et al., 2025). The most common form involves compression of the LRV within the aortomesenteric angle formed by the abdominal aorta (AO) and the superior mesenteric artery (SMA). With advancing clinical understanding, several anatomical variants have been identified, including anterior and posterior NCS, as well as other rare forms. The syndrome was first denominated by Schepper in 1972 (De, 1972). The compressed LRV undergoes marked morphological alterations, including reduced cross-sectional area and consequent hemodynamic disturbances (Barnes et al., 1988; Granata et al., 2021). When these anatomical abnormalities are accompanied by clinical symptoms such as left flank or abdominal pain, hematuria, and proteinuria, the condition is referred to as nutcracker syndrome (Cope and Isard, 1969; Orczyk et al., 2017).

Anatomically, the left renal vein is more complex than its right counterpart, with a mean length of approximately 8.5 cm (Bowdino et al., 2026). Along its course, it receives multiple tributaries, including the adrenal, inferior phrenic, lumbar, and gonadal veins (Shreevastava et al., 2024; Yi et al., 2012). This intricate venous network serves a significant compensatory role in LRV obstruction: its tributaries and anastomotic channels can dilate and enlarge, forming rich collateral drainage pathways (Kurklinsky and Rooke, 2010). As a major tributary of the LRV, the gonadal vein occupies a critical position within this venous drainage system.

In male patients with NCS, compression of the LRV frequently involves its major branch—the gonadal vein—leading to impaired gonadal venous return and subsequent varicocele (Scultetus et al., 2001). Varicocele is a well-established contributor to male infertility, clinically presenting as scrotal heaviness or pain and potentially leading to impaired testicular function (Kim et al., 2025). Studies indicate that the incidence of varicocele in NCS patients ranges from 40% to 60%, significantly higher than that in the general population (Orczyk et al., 2016; Penfold et al., 2026). Therefore, a thorough understanding of the pathophysiological mechanisms underlying NCS-associated varicocele is essential for improving patient quality of life and fertility outcomes. Computational fluid dynamics (CFD) has emerged as a powerful non-invasive tool for investigating hemodynamics in vascular diseases, offering high-resolution hemodynamic simulation capabilities (Shimodoumae et al., 2024). In recent years, CFD has been increasingly applied to study various vascular conditions, including aneurysms, arterial stenosis, and venous compression syndrome (Philip and Patnaik, 2021; Mu et al., 2021). Research (Chen et al., 2025) utilized CT imaging data from two NCS patients to construct three-dimensional models of the LRV. Their study was the first to employ CFD to characterize the abnormal distributions of pressure, velocity, and wall shear stress within the LRV, demonstrating that increased severity of compression exacerbates flow disturbance and vascular damage. However, this study did not address the common NCS complication of varicocele, nor did it simulate the impact of the clinically critical Valsalva maneuver on hemodynamics.

The Valsalva maneuver, a physiological operation involving forced expiration against a closed glottis that increases intrathoracic and intra-abdominal pressure, is routinely employed in ultrasound diagnosis of varicocele to induce or accentuate venous reflux (You et al., 2023). Clinical guidelines recommend that patients maintain the Valsalva maneuver for 4–6 s to adequately assess the degree of reflux (Freeman et al., 2020). The maneuver significantly elevates intra-abdominal venous pressure, further compressing the LRV and causing a sharp increase in pressure at the gonadal vein origin, thereby inducing or exacerbating gonadal venous reflux. Notably, this mechanical effect is not confined to conscious Valsalva maneuvers; strenuous daily activities such as running, jumping, and heavy lifting can also induce periodic or sustained elevations in intra-abdominal pressure. Previous studies have demonstrated that running can transiently increase intra-abdominal pressure to 30–50 mmHg, approximating some effects of the Valsalva maneuver (Grillner et al., 1978). For NCS patients, whose LRV is already in a state of compression, exercise-induced increases in intra-abdominal pressure may further aggravate LRV compression, leading to repeated fluctuations in gonadal vein inlet pressure and consequently provoking or exacerbating venous reflux. This phenomenon suggests that recurrent intra-abdominal pressure elevations during daily activities may periodically exacerbate LRV compression and contribute to the clinical presentation of post-exercise scrotal discomfort in NCS patients with varicocele. These observations underscore the potential clinical value of incorporating patients’ daily activity patterns and dynamic hemodynamic changes into disease assessment. However, to date, no study has quantitatively characterized the hemodynamic response of the gonadal vein and LRV throughout a complete Valsalva cycle. This knowledge gap limits our understanding of the pathophysiology of NCS-associated varicocele and constrains the development of individualized treatment strategies.

The present study addresses these gaps by constructing a three-dimensional model incorporating the gonadal vein and applying a time-varying pressure boundary condition at its inlet to simulate Valsalva. Using CFD, we systematically analyze pressure, velocity, wall shear stress, and streamline distributions within the gonadal vein and LRV throughout the complete Valsalva cycle. This approach aims to elucidate the hemodynamic mechanism by which Valsalva exacerbates varicocele and to advance the clinical translation of CFD in venous disorders. The findings are expected to deepen understanding of NCS pathophysiology and inform precision diagnosis and treatment strategies.

## Methods

### Study population and data acquisition

This retrospective study consecutively enrolled 57 patients diagnosed with nutcracker syndrome (NCS) and concomitant varicocele at Tangdu Hospital between December 2020 and December 2025. Inclusion criteria were as follows: (1) confirmed left renal vein (LRV) compression on computed tomography angiography (CTA), defined as an aortomesenteric angle <35° or a stenotic-to-inlet diameter ratio <0.5; (2) demonstrated gonadal venous reflux on Doppler ultrasonography; and (3) availability of complete clinical and imaging data. Patients were excluded if they met any of the following criteria: (1) history of abdominal or vascular surgery; (2) presence of other concomitant venous disorders; or (3) insufficient image quality for accurate measurement.

All patients underwent standardized Doppler ultrasonography, performed independently by two experienced sonographers. The following parameters were measured: aortomesenteric angle, LRV diameter, minimal diameter at the stenotic segment, peak velocity at the stenosis, LRV inlet velocity, and the diameter of the gonadal vein inlet under both resting conditions and during the Valsalva maneuver. Each parameter was measured three times (Table 1). The study protocol was approved by the Institutional Review Board of Tangdu Hospital, and informed consent was obtained from all participants.

**TABLE 1:** Baseline characteristics of study participants.

Parameter	Value (mean ± SD)
Aortomesenteric angle (°)	19.83 ± 5.53
Left renal vein diameter (mm)	10.06 ± 2.74
Minimal diameter at stenosis (mm)	1.64 ± 0.73
Stenotic-to-inlet diameter ratio	∼0.16
Peak velocity at stenosis (cm/s)	127.21 ± 45.22
LRV inlet velocity (cm/s)	13.78 ± 3.93
Stenotic-to-inlet velocity ratio	∼9.2
Gonadal vein diameter at rest (mm)	3.0 ± 0.94
Gonadal vein diameter during Valsalva (mm)	3.86 ± 1.07

### Three-dimensional geometric model reconstruction

**FIGURE 1:**
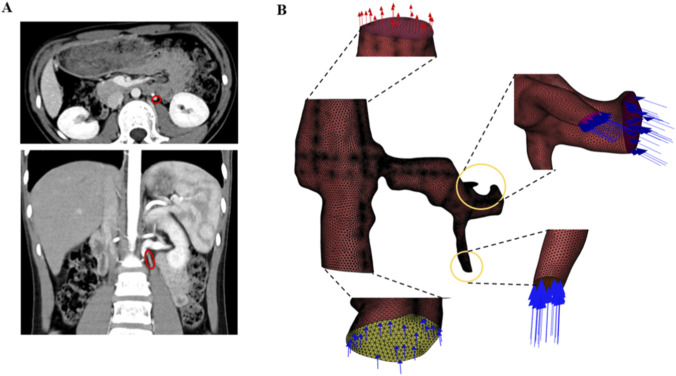
**(A)** Contrast-enhanced CT from a representative patient. **(B)** Tetrahedral three-dimensional mesh of the geometric model.

A representative three-dimensional geometric model was reconstructed from contrast-enhanced CT angiography data of a patient with typical NCS anatomy (aortomesenteric angle: 20°; minimal LRV diameter at stenosis: 2.5 mm). Segmentation and surface reconstruction were performed using 3D Slicer software (version 4.11, Brigham and Women’s Hospital, Boston, MA, United States). The computational domain encompassed the inferior vena cava (IVC), the left renal vein (LRV), and the gonadal vein (GV), as illustrated in Figure 1A. To ensure computational accuracy, regions with high curvature were smoothed, and the model was exported in STL format for subsequent mesh generation.

### Mesh generation and independence study

The computational domain was discretized using tetrahedral meshes in ANSYS ICEM CFD (ANSYS, Inc., Canonsburg, PA, United States). Local mesh refinement was applied to regions of interest, including the stenotic segment, vascular bifurcations, and vicinities near the inlet and outlet boundaries (Figure 1B). Mesh quality was assessed by orthogonal quality and skewness metrics; a minimum orthogonal quality >0.15 and a maximum skewness <0.85 were considered acceptable for CFD simulations.

A grid independence study was conducted using four mesh densities (approximately 0.3, 0.4, 0.5, and 0.6 million elements). Velocity and pressure at the stenosis center (monitoring point P_2_) were compared. The differences between the 0.4-million and 0.5-million grids were less than 2%, indicating grid independence.

### Boundary conditions and numerical setup

Blood was modeled as an incompressible Newtonian fluid with density ρ = 1056 kg/m^3^ and dynamic viscosity μ = 0.0035 Pa·s. The flow was assumed to be laminar and transient, governed by the three-dimensional incompressible Navier–Stokes equations:

$$\begin{array}{c} \nabla \cdot u=0\\ \frac{\partial u}{\partial t}+\left(u\cdot \nabla \right)u=-\frac{1}{\rho }\nabla p+\nu \nabla^{2}u \end{array}$$

where u is the velocity vector, p is the pressure, andν = μ/ρ is the kinematic viscosity.

Inlet boundary conditions: The LRV inlet velocity was prescribed based on the mean value derived from Doppler measurements of the 57 patients (13.78 ± 3.93 cm/s). To account for measurement variability and maintain consistency with the approach of Chen et al. (Chen et al., 2025), the mean value of 0.5 m/s (converted from 50 cm/s) was adopted. The GV inlet was treated as a pressure outlet (see below). All other inlets (adrenal, lumbar, etc.) were assumed to have negligible flow and were modeled as no-slip walls.

Outlet boundary conditions: The IVC outlet was set as a pressure outlet with a constant pressure of 100 mmH_2_O, representing typical venous pressure.

**FIGURE 2:**
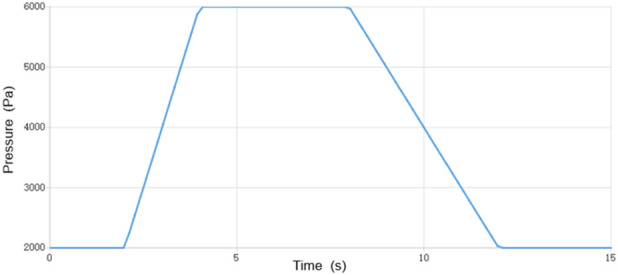
Time-varying pressure boundary condition at the gonadal vein inlet during Valsalva maneuver.

Valsalva maneuver simulation: To replicate the hemodynamic impact of the Valsalva maneuver, a time-varying pressure boundary condition was applied at the GV inlet over a 12-s cycle, as depicted in Figure 2. The pressure curve consisted of four distinct phases: (i) resting baseline; (ii) ascending phase; (iii) plateau phase; and (iv) recovery phase (Laborda et al., 2014). This design ensures physiological relevance and allows quantitative assessment of hemodynamic responses. Wall conditions: All vessel walls were assumed rigid with no-slip boundary conditions.

### Numerical solution

Simulations were performed using ANSYS Fluent 2021 R1 (ANSYS, Inc.). The coupled algorithm was employed for pressure–velocity coupling, with second-order upwind spatial discretization and second-order implicit temporal discretization. A fixed time step of 0.01 s was used, yielding 1200 time steps per Valsalva cycle. Convergence criteria were set to residuals <10^–5^ for all equations. Each simulation required approximately 8 h on a workstation with an Intel Xeon Gold 5218 CPU and 128 GB RAM.

Hemodynamic parameters and data extraction.

Hemodynamic parameters analyzed included pressure, velocity magnitude, wall shear stress (WSS), and streamline patterns. WSS was computed as:

$$\text{WSS}=Tn-\left(Tn\cdot n\right)n$$

where Tn is the wall traction vector and n is the unit normal to the wall. Three monitoring points were placed along the LRV–GV pathway: P_1_ (proximal LRV, near the renal hilum), P_2_ (center of the stenosis), and P_3_ (downstream of the stenosis, near the GV origin). Time histories of pressure and velocity at these points were recorded.

### Statistical analysis

Continuous variables are presented as mean ± standard deviation. Comparisons between resting and Valsalva conditions for GV diameter were performed using paired t-tests. A p-value <0.05 was considered statistically significant. All statistical analyses were conducted using SPSS version 26.0 (IBM Corp., Armonk, NY, United States).

## Results

A total of 57 patients with NCS and concomitant varicocele were enrolled in this study. The baseline clinical and ultrasonographic characteristics of the study population are summarized in Table 1.

Using contrast-enhanced CT data from a representative patient, we reconstructed the three-dimensional model with 3D Slicer software, as shown in Figure 1A. The three-dimensional computational domain constructed in this study comprises the geometric models of the inferior vena cava, left renal vein, and gonadal vein, which were discretized using tetrahedral meshes. To ensure accurate computation of hemodynamic parameters, local mesh refinement was applied to regions of interest, including the stenotic segment, vascular bifurcations, and vicinities near the inlet and outlet boundaries. As shown in Figure 1B, the left renal vein exhibits pronounced compression and deformation as it traverses the narrow space between the superior mesenteric artery and the abdominal aorta, resulting in a substantial reduction in its cross-sectional area. Mesh quality assessment yielded a minimum orthogonal quality greater than 0.15 and a maximum skewness below 0.85, both satisfying the standard requirements for CFD simulations. The resulting model provides a reliable geometric foundation for subsequent precise hemodynamic analysis of the gonadal vein during the Valsalva maneuver.

To accurately replicate the hemodynamic impact of the Valsalva maneuver, a physiologically relevant time-varying pressure boundary condition was prescribed at the gonadal vein inlet over a 12-s cycle, as depicted in Figure 2. This pressure curve consists of four distinct phases: a resting baseline phase, an ascending phase (2–4 s), a plateau phase (4–8 s), and a recovery phase (8–12 s).

The design of this pressure profile was based on standard clinical Doppler ultrasonography protocols, which require patients to sustain the Valsalva maneuver for 4–6 s to effectively provoke venous reflux, thereby faithfully replicating the physiological pressurization effect of the Valsalva maneuver on the abdominal venous system. This carefully defined boundary condition ensures that the simulation results accurately capture the hemodynamic response of the gonadal vein during the Valsalva maneuver.

**FIGURE 3:**
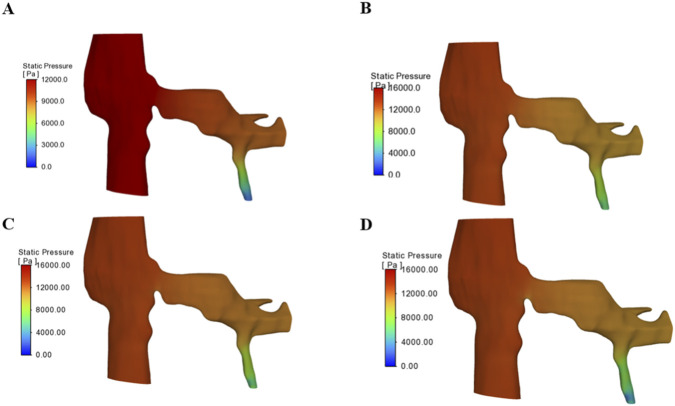
Pressure distribution contours within the left renal vein 4 and gonadal vein at four representative time points during the Valsalva maneuver. **(A)** Resting baseline phase, t = 2 s; **(B)** Ascending phase, t = 4 s; **(C)** Plateau phase, t = 6 s; **(D)** Recovery phase, t = 10 s.

The pressure distribution contours within the left renal vein (LRV) and gonadal vein were analyzed at four representative time points during the Valsalva maneuver (t = 2, 4, 6, and 10 s), as shown in Figures 3A–D. At the resting baseline phase (t = 2 s), the pressure in the proximal segment of the LRV remained within the range of 2000–2666 Pa. A slight pressure reduction was observed downstream of the stenotic site, while the overall pressure gradient remained relatively flat, consistent with the characteristic venous hypertension typically observed in NCS patients under resting conditions (Figure 3A). Upon initiation of the Valsalva maneuver (t = 4 s), the pressure at the gonadal vein inlet rapidly increased to 6,000 Pa. This pressure wave propagated upstream into the LRV, resulting in a concomitant elevation of proximal LRV pressure to above 4,500 Pa. A marked increase in the pressure gradient across the stenotic segment was evident, characterized by a pronounced contrast between high upstream and low downstream pressures (Figure 3B). During the plateau phase (t = 6 s), with sustained Valsalva, the pressure at the gonadal vein inlet stabilized at 6,000 Pa, while the pressure upstream of the LRV stenosis peaked at approximately 5,000 Pa. Simultaneously, a distinct low-pressure region developed downstream of the stenosis. This configuration generated a substantial pressure gradient of approximately 7,600 Pa across the stenotic segment, thereby significantly increasing the compressive load on the stenotic site (Figure 3C). Following release of the Valsalva maneuver during the recovery phase (t = 10 s), pressures in both the LRV and gonadal vein rapidly returned to baseline levels. The low-pressure region downstream of the stenosis disappeared, and the pressure distribution resumed a more uniform pattern (Figure 3D).

**FIGURE 4:**
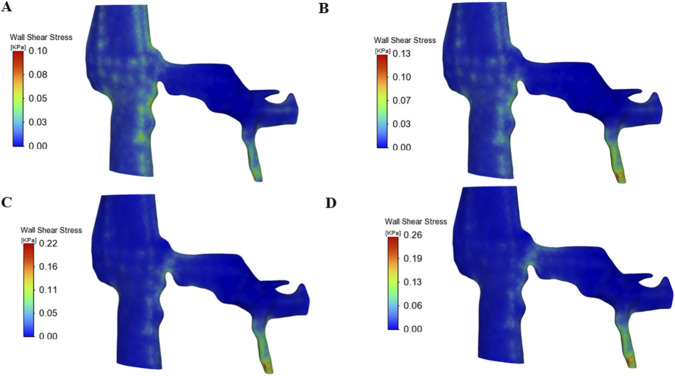
Distribution of wall shear stress within the left renal vein and gonadal vein at four representative time points during the Valsalva maneuver. **(A)** Resting baseline phase, t = 2 s; **(B)** Ascending phase, t = 4 s; **(C)** Plateau phase, t = 6 s; **(D)** Recovery phase, t = 10 s.

Figure 4 illustrates the distribution of wall shear stress (WSS) on the inner wall of the left renal vein (LRV) and gonadal vein at four representative time points during the Valsalva maneuver. At the resting baseline phase (A, t = 2 s), WSS was predominantly low across the LRV and its branches, with most regions exhibiting values below 100 Pa. A mild elevation of WSS was observed at the stenotic site of the LRV, while the gonadal vein inlet showed only minimal, localized increases in shear stress, maintaining an overall low hemodynamic load on the vascular endothelium. During the ascending phase (B, t = 4 s), accelerated blood flow through the stenotic segment drove a rapid rise in WSS, with the maximum value at the stenosis reaching 130 Pa. Concurrently, a marked elevation in WSS was evident near the gonadal vein inlet, with localized high-shear regions extending along the vessel wall, indicating intensified frictional forces acting on the endothelial layer. At the plateau phase (C, t = 6 s), WSS reached its peak magnitude across the vascular geometry: the maximum WSS at the stenotic center attained 220 Pa, increase compared to the resting baseline. Elevated WSS regions were predominantly concentrated on the upstream lateral wall of the stenotic segment and the proximal segment of the gonadal vein, exhibiting pronounced spatial heterogeneity in shear stress distribution. Following release of the Valsalva maneuver at the recovery phase (D, t = 10 s), WSS rapidly decreased but remained elevated relative to baseline, with the maximum value at the stenosis and gonadal vein inlet reaching 260 Pa in the late recovery period, before gradually returning to resting levels.

**FIGURE 5:**
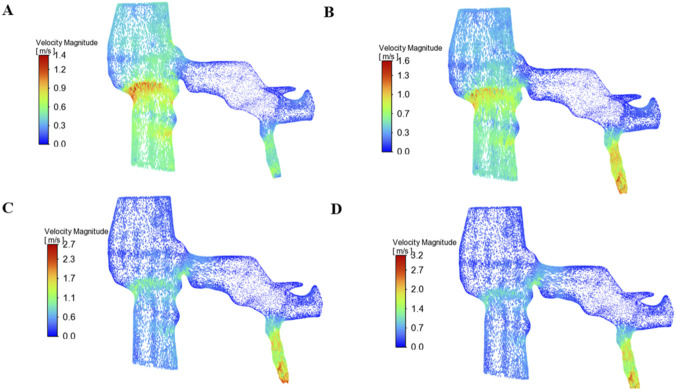
Velocity and streamline distributions within the left renal vein and gonadal vein at four representative time points during the Valsalva maneuver. The visualization is a three-dimensional view; for anatomical reference, the long axes of the left renal vein and gonadal vein lie approximately in the coronal plane. **(A)** Resting baseline phase, t = 2 s; **(B)** Ascending phase, t = 4 s; **(C)** Plateau phase, t = 6 s; **(D)** Recovery phase, t = 10 s.

The velocity contours and streamline distributions within the left renal vein (LRV) and gonadal vein were analyzed at four representative time points during the Valsalva maneuver, as depicted in Figures 5A–D. At the resting baseline phase (t = 2 s), blood flow exhibited a uniform distribution within the proximal LRV, with most regions showing velocities below 0.6 m/s and smooth, laminar streamlines. A modest flow acceleration was observed at the stenotic site, with a peak velocity reaching approximately 1.4 m/s; however, no significant flow separation or recirculation was evident at this stage, and flow in the gonadal vein remained sluggish with low velocity (Figure 5A). During the ascending phase (t = 4 s), the initiation of the Valsalva maneuver induced a rapid elevation in velocity at the stenosis, with the maximum value reaching 1.6 m/s, accompanied by a marked increase in flow velocity at the gonadal vein inlet, where localized high-velocity regions emerged. Streamlines became condensed within the stenotic segment, with incipient flow disturbance observed downstream of the stenosis, indicating early hemodynamic perturbation (Figure 5B). At the plateau phase (t = 6 s), peak velocity was attained, with the maximum velocity at the stenotic center reaching 2.7 m/s, representing a near-doubling of the baseline peak velocity. Streamlines exhibited extreme condensation within the stenotic region, and a pronounced high-velocity jet formed at the gonadal vein inlet, with peak velocities exceeding 2.3 m/s. Pronounced flow separation and disturbed streamlines were observed downstream of the stenosis, while the gonadal vein demonstrated sustained high-velocity outflow, exacerbating venous hemodynamic load (Figure 5C). Following release of the Valsalva maneuver during the recovery phase (t = 10 s), velocity at the stenosis remained elevated at 3.2 m/s in the late recovery period, with the gonadal vein inlet still exhibiting high-velocity flow before gradually returning to baseline levels. Recirculation zones gradually dissipated, streamlines resumed smooth laminar configurations, and flow conditions ultimately reverted to patterns characteristic of the resting state (Figure 5D). The dramatic velocity surge at the stenotic site and gonadal vein inlet during the Valsalva maneuver, coupled with the sustained high-flow disturbance, represents a direct hemodynamic driver of exacerbated venous reflux in nutcracker syndrome (NCS)-associated varicocele. To assess the generalizability of the observed hemodynamic cycle across different anatomical configurations, two additional patient-specific models were constructed based on CT imaging data from independent NCS patients with varying degrees of left renal vein compression (Model A and Model B; see Supplementary Figures S1–S6). These findings confirm that the qualitative hemodynamic patterns described in the main text are robust across patients with different degrees of compression, supporting the generalizability of the proposed pathophysiological mechanism.

## Discussion

The Valsalva maneuver is routinely employed in clinical ultrasound diagnosis to induce or accentuate gonadal venous reflux. Its physiological essence involves forced expiration against a closed glottis, resulting in significant elevation of intrathoracic and intra-abdominal pressure (Kim et al., 2015). From the perspective of hemodynamic research, the value of the Valsalva maneuver extends beyond its role as a diagnostic procedure. It provides a standardized, quantifiable, and reproducible physiological model. This model simulates intra-abdominal pressure elevations induced by various causes in daily life. In daily activities, transient or sustained elevations in intra-abdominal pressure are exceedingly common: running can transiently increase intra-abdominal pressure to 30–50 mmHg, while activities such as jumping, heavy lifting, coughing, and defecation can also induce varying degrees of intra-abdominal pressure fluctuations. In healthy individuals, these physiological pressure fluctuations are adequately buffered by the compensatory mechanisms of the venous system. However, in patients with NCS, the LRV is already in a state of compression, and its compensatory capacity is consequently compromised. Therefore, this study employed the Valsalva maneuver as a standardized simulation model for daily intra-abdominal pressure elevations. By applying a time-varying pressure boundary condition, we systematically elucidated the hemodynamic impact of intra-abdominal pressure elevation on the gonadal vein in NCS patients.

The results of this study demonstrate that during the plateau phase of simulated intra-abdominal pressure elevation, the pressure at the gonadal vein inlet increased to 6,000 Pa, the velocity at the stenosis surged to more than five times the inlet velocity, and the peak wall shear stress reached 260 Pa, representing a 12-fold increase compared to the resting baseline. These findings quantitatively characterize the mechanical load imposed on the gonadal vein by intra-abdominal pressure elevation and provide a mechanistic explanation for the clinical phenomenon of exacerbated varicocele reflux. Notably, this study revealed that intra-abdominal pressure elevation induces a substantial pressure gradient across the LRV stenosis, with a concurrent high-pressure region upstream and a negative-pressure region downstream. This high-pressure-negative-pressure configuration exerts an inwardly directed net force on the vascular wall at the stenotic segment, leading to further collapse and thereby exacerbating the degree of compression. Increased compression, in turn, further widens the upstream-downstream pressure gradient, establishing a self-perpetuating positive feedback loop of high-pressure-negative-pressure-recompression. This mechanism provides an explanation for two important clinical features of NCS. First, progressive worsening of symptoms: even with initially mild compression, recurrent intra-abdominal pressure elevations during daily activities (e.g., running, jumping) may lead to cumulative hemodynamic deterioration. This results in progressive symptom exacerbation over time. Second, activity-related symptom fluctuation: the scrotal discomfort or pain experienced by patients after activities that elevate intra-abdominal pressure (such as running or heavy lifting) may represent the transient initiation of this vicious cycle.

Wall shear stress, the frictional force exerted by blood flow on the vascular endothelium, is a critical biomechanical regulator of endothelial function, vascular remodeling, and thrombosis (Tarbell et al., 2014). The present study found that during the plateau phase of simulated intra-abdominal pressure elevation, the peak WSS at the stenosis reached 260 Pa. Such extreme elevations in WSS can directly induce endothelial denudation, basement membrane exposure, and platelet aggregation. These effects may potentially trigger thrombosis and vascular wall remodeling. From a clinical perspective, NCS patients are repeatedly exposed to intra-abdominal pressure elevations during daily activities, implying that their vascular endothelium may be subjected to cumulative mechanical injury. Each foot strike during running, each impact during jumping, may transiently induce a sharp increase in WSS. While a single such event may be inconsequential, the cumulative effect of repetitive stimulation over time may lead to progressive endothelial dysfunction, ultimately manifesting as worsening clinical symptoms and the development of complications. Furthermore, this study observed pronounced spatial heterogeneity in WSS distribution—regions of elevated WSS were predominantly concentrated on the upstream lateral wall of the stenotic segment and in the vicinity of the gonadal vein inlet. Such spatial gradients can, in themselves, activate endothelial mechanoreceptors, inducing localized inflammatory responses and expression of matrix metalloproteinases, thereby contributing to vascular wall weakening (Mu et al., 2022). This observation provides a mechanistic basis for understanding why vascular lesions in NCS often exhibit a focal rather than diffuse distribution.

This study demonstrated that during the plateau phase of simulated intra-abdominal pressure elevation, stable recirculation zones developed downstream of the stenosis. Within these recirculation zones, flow velocities were extremely low or exhibited reversed direction, thereby impeding effective blood entry into the inferior vena cava and reducing effective flow volume by approximately 50%–60%. This flow pattern is highly consistent with the hemodynamic disturbances reported by Hansraj et al. in cases of post-stent restenosis in NCS patients.

The formation of recirculation zones carries multiple pathophysiological implications: First, it compromises venous return efficiency—recirculation zones occupy 30%–40% of the vascular cross-sectional area, reducing the effective flow area, increasing flow resistance, and further aggravating upstream venous hypertension. Second, it promotes thrombogenesis—stagnant flow and low WSS within recirculation zones constitute one of the classic elements of Virchow’s triad. Third, it exacerbates vascular wall injury—high WSS gradients exist at the boundaries of recirculation zones, and the reciprocating impact of flow within these zones subjects the endothelium to repetitive mechanical stimulation.

Interpreted within the context of daily activities, these findings imply that for NCS patients, each episode of intra-abdominal pressure elevation during activities such as running or jumping may transiently induce recirculation zone formation. This leads to a temporary reduction in flow efficiency. Although flow conditions may return to normal at rest, the repetitive ischemia-reperfusion-like pattern may inflict cumulative injury on the vascular wall.

The findings of this study are highly consistent with those of Chen et al. (2025), who modeled the LRV in isolation. Both studies observed a 4- to 5-fold increase in velocity at the stenosis, a 15- to 20-fold increase in WSS, and a 50%–60% reduction in effective flow volume due to recirculation zone formation. This consistency validates the computational methods and the reliability of the results presented herein. However, the present study further reveals that the gonadal vein, as a branch of the LRV, experiences a more pronounced mechanical load under conditions of elevated intra-abdominal pressure. The magnitude of pressure increase at the gonadal vein inlet exceeded that at the LRV inlet. Additionally, the WSS near the gonadal vein inlet during the plateau phase reached more than 80% of the peak WSS at the LRV stenosis. These findings suggest that the gonadal vein is not merely a passively affected vessel in NCS. Rather, it represents a critical site of hemodynamic abnormality during intra-abdominal pressure elevation.

The findings of this study have multiple implications for the clinical management of NCS-associated varicocele: At the diagnostic level, this study suggests that hemodynamic assessment performed solely under resting conditions may underestimate the true severity of disease in NCS patients. Given that patients’ symptoms typically manifest or worsen following activity, dynamic hemodynamic assessment—i.e., measurements obtained under conditions that simulate or induce intra-abdominal pressure elevation—should constitute an integral component of clinical evaluation. The parameters identified in this study under conditions of elevated intra-abdominal pressure, including pressure gradients, peak WSS, and recirculation zone area, may serve as novel quantitative diagnostic indicators for NCS-associated varicocele. At the therapeutic level, the high-pressure-negative-pressure-recompression vicious cycle elucidated in this study provides clear hemodynamic targets for extravascular stent design. An ideal stent must be capable of withstanding recurrent intra-abdominal pressure elevations encountered during daily activities while preserving patency of the stenotic segment under dynamic physiological conditions. From a clinical perspective, the present findings suggest that patients with NCS-associated varicocele should be counseled regarding the potential impact of daily activities that elevate intra-abdominal pressure on disease progression. While complete avoidance of activities such as running or jumping may be impractical, patients are advised to adopt modified exercise patterns—selecting activities associated with lower intra-abdominal pressure fluctuations—and to allow adequate rest during symptomatic exacerbations, thereby mitigating the risk of cumulative mechanical injury.

This study did not incorporate venous valves, vascular wall elasticity, or pulsatile flow in the inferior vena cava. The absence of venous valves in our model represents a limitation, as valve incompetence is a core pathological feature of varicocele. In the physiological state, competent valves prevent retrograde flow and disrupt recirculation zone formation. In patients with varicocele, valve incompetence allows retrograde flow to propagate from the LRV into the gonadal vein during Valsalva. This pathological mechanism likely interacts synergistically with the recirculation zones identified in our model, amplifying the volume of stagnant or reversed flow and exacerbating endothelial injury. The observed flow patterns are consistent with the pathognomonic finding of Valsalva-induced reflux on Doppler ultrasonography, and future models incorporating valve structures would further clarify the interplay between valvular dysfunction and flow separation phenomena. Venous valves play a critical role in gonadal venous reflux and should be included in future models. Vascular elasticity may buffer pressure fluctuations and influence negative pressure formation; subsequent studies should employ fluid-structure interaction methods to more realistically simulate vascular responses to recurrent pressure fluctuations during daily activities. Several technical limitations should be acknowledged. First, the assumption of rigid vessel walls may lead to overestimation of peak velocity and wall shear stress values. Under physiological conditions, compliant veins undergo compensatory dilation during Valsalva, which would attenuate flow acceleration and reduce peak WSS. Therefore, the absolute WSS values reported likely represent upper-bound estimates. However, the directional changes and qualitative flow patterns—including the formation of recirculation zones and the development of pressure gradients—are expected to be robust. Second, the inferior vena cava outlet was modeled with a constant pressure boundary condition, which simplifies the dynamic pressure fluctuations that occur during Valsalva. While the IVC is downstream of the LRV and its pressure fluctuations are dampened relative to upstream veins, a time-varying IVC boundary condition would more accurately reflect the physiological state. However, the pressure gradient across the LRV stenosis—the primary driver of the observed hemodynamic changes—is predominantly influenced by the pressure imposed at the gonadal vein inlet. Future studies incorporating dynamic IVC boundary conditions could further refine the quantitative assessment of trans-stenotic pressure gradients. While this study employed the Valsalva maneuver as a standardized simulation model for daily intra-abdominal pressure elevations, the patterns of intra-abdominal pressure variation during actual daily activities are considerably more complex and diverse. These include rhythmic fluctuations during running and sustained elevations during heavy lifting. Based on the foundation established in this study, future research directions may include developing fluid-structure interaction models that incorporate venous valves and vascular wall elasticity to simulate cumulative injury from recurrent pressure fluctuations during daily activities. Clinical studies of dynamic hemodynamics are also warranted, with measurements of flow parameters before and after actual activities such as running or lifting. Additionally, CFD-based optimization platforms for individualized extravascular stent design should be developed to ensure that stents can withstand the repetitive mechanical loads encountered during daily life. Finally, the potential value of lifestyle interventions, such as exercise modification, should be investigated in slowing disease progression.

## Conclusion

This study employing the Valsalva maneuver as a standardized simulation model for daily intra-abdominal pressure elevations, is the first to systematically elucidate, using CFD methods, the hemodynamic response characteristics of the gonadal vein in NCS patients under conditions of elevated intra-abdominal pressure. The main conclusions are as follows: Intra-abdominal pressure elevation induces a substantial pressure gradient across the LRV stenosis, initiating a self-perpetuating high-pressure-negative-pressure-recompression vicious cycle. Wall shear stress increases more than 12-fold under conditions of elevated intra-abdominal pressure, exhibits marked spatial heterogeneity, and represents a key mechanical factor contributing to cumulative endothelial injury. Velocity surge and recirculation zone formation lead to a 50%–60% reduction in effective flow volume, and flow stagnation within recirculation zones may promote thrombogenesis. Recurrent intra-abdominal pressure elevations during daily activities may result in cumulative mechanical injury, providing an explanation for the clinically observed phenomena of progressive symptom worsening and activity-related symptom exacerbation in NCS patients. These findings advance the understanding of the pathophysiological mechanisms underlying NCS-associated varicocele and provide a scientific foundation for dynamic hemodynamic assessment, optimization of individualized treatment strategies, and evidence-based patient guidance regarding lifestyle modifications.

## Data Availability

The original contributions presented in the study are included in the article/Supplementary Material, further inquiries can be directed to the corresponding authors.
