# Supplementary material for: Numerical simulation of gonadal vein hemodynamics in nutcracker syndrome-associated varicocele: effects of Valsalva maneuver and clinical implications
Source: Front Bioeng Biotechnol. 2026 May 18;14:1828724. doi: 10.3389/fbioe.2026.1828724 (PMC13223007; doi:10.3389/fbioe.2026.1828724)
Supplement: Supplementary file 1 [file Supplementaryfile1.docx]

To assess the generalizability of the observed hemodynamic cycle across different anatomical configurations, two additional patient-specific models (Model A and Model B) were constructed based on CT imaging data from independent NCS patients with varying degrees of left renal vein compression. Figures S1–S3 present the simulation results for Model A (mild‑to‑moderate compression), and Figures S4–S6 present the results for Model B (moderate‑to‑severe compression). For each model, pressure distribution contours (Fig. S1 and S4), wall shear stress distribution (Fig. S2 and S5), and velocity/streamline distributions (Fig. S3 and S6) are shown at four representative time points during the Valsalva maneuver (t = 2, 4, 6, and 10 s), corresponding to the resting baseline, ascending, plateau, and recovery phases, respectively.

Consistent with the original representative model, both Model A and Model B exhibited the characteristic “high‑pressure‑negative‑pressure” pattern across the left renal vein stenosis during the Valsalva plateau phase (t = 6 s), with marked elevation of wall shear stress and the development of stable recirculation zones downstream of the stenosis, accompanied by a 45%–65% reduction in effective flow volume. These findings confirm that the qualitative hemodynamic patterns described in the main text are robust across patients with different degrees of compression, supporting the generalizability of the proposed pathophysiological mechanism**.**

**
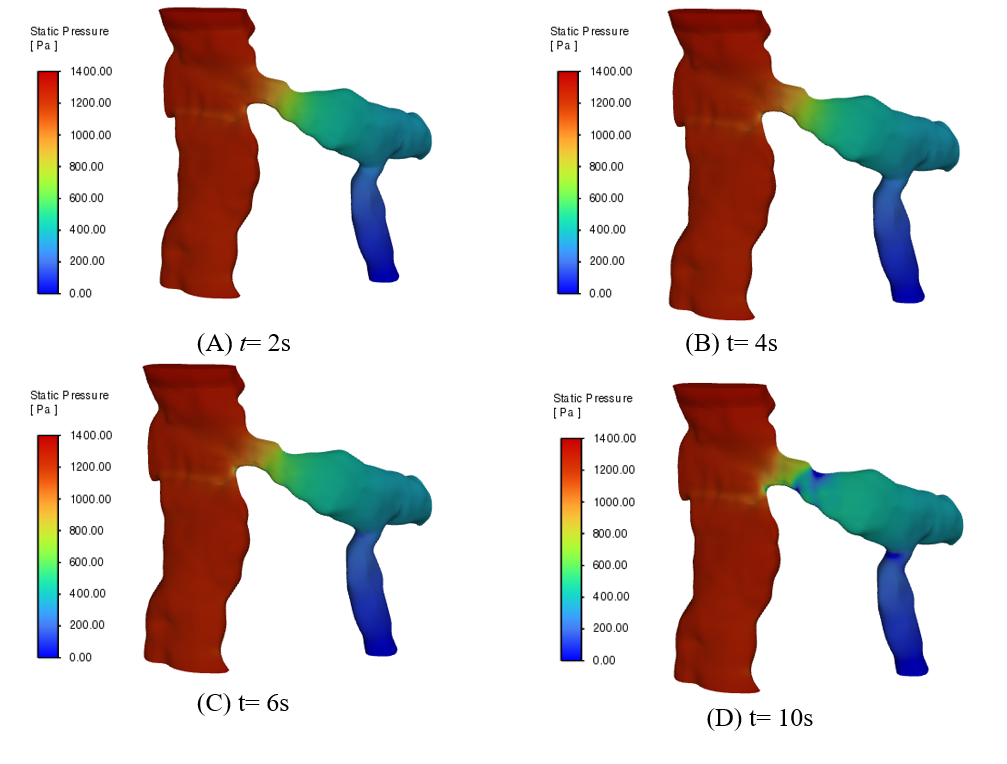
**

**Fig. S1**. Pressure distribution contours within the left renal vein 4·and gonadal vein at four representative time points during the Valsalva maneuver (A) Resting baseline phase, t = 2 s; (B) Ascending phase, t = 4 s; (C) Plateau phase, t = 6 s; (D) Recovery phase, t = 10 s


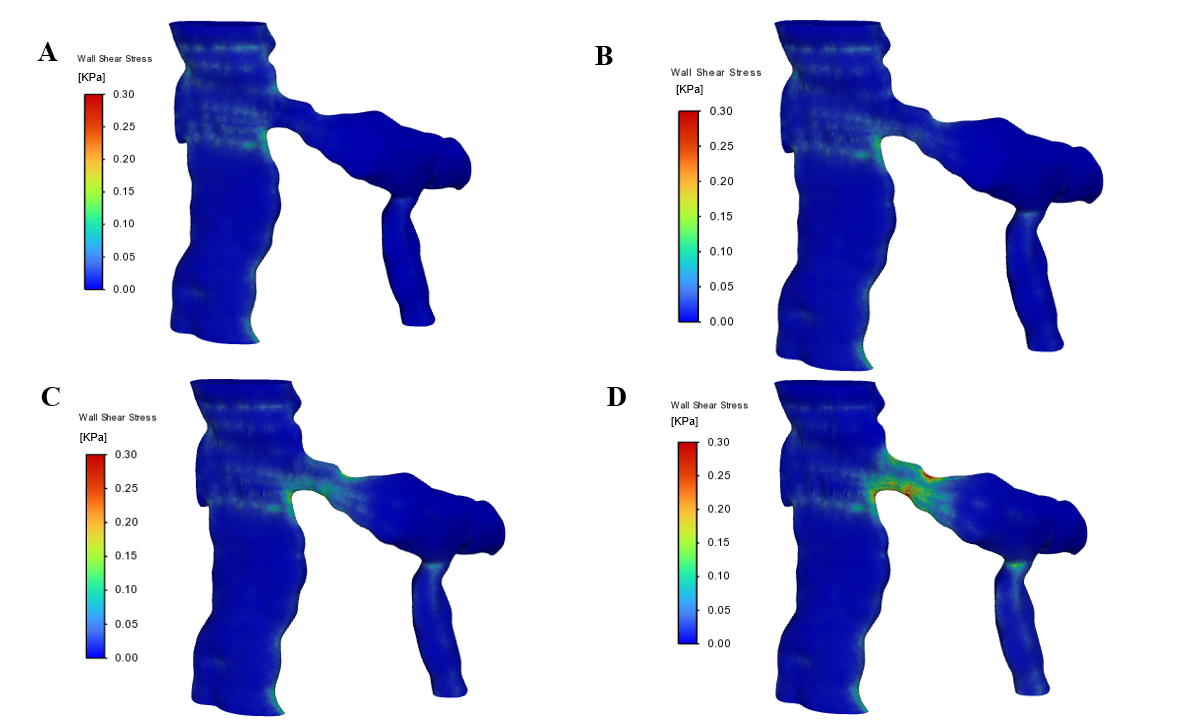


**Fig. S2**. Distribution of wall shear stress within the left renal vein and gonadal vein at four representative time points during the Valsalva maneuver (A) Resting baseline phase, t = 2 s; (B) Ascending phase, t = 4 s; (C) Plateau phase, t = 6 s; (D) Recovery phase, t = 10 s

| 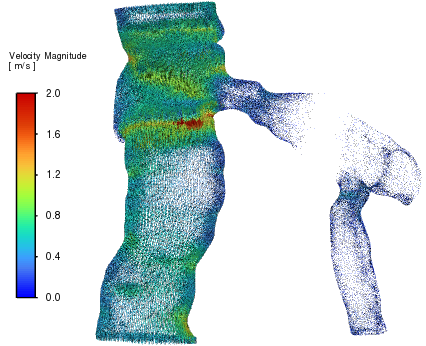 | 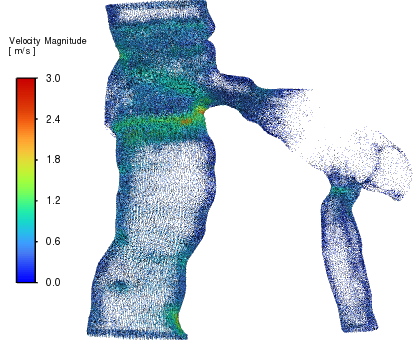 |
| --- | --- |
| (A) *t*= 2s | (B) *t*= 4s |
| 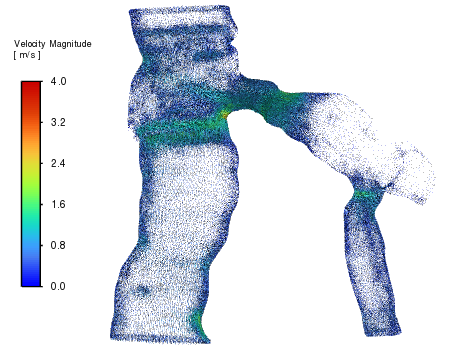 | 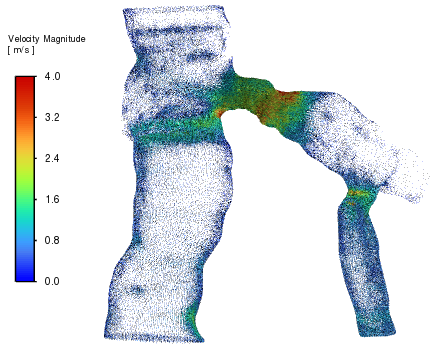 |
| (C) *t*= 6s | (D) *t*= 10s |

**Fig. S3**. Velocity and streamline distributions within the left renal vein and gonadal vein at four representative time points during the Valsalva maneuver (A) Resting baseline phase, t = 2 s; (B) Ascending phase, t = 4 s; (C) Plateau phase, t = 6 s; (D) Recovery phase, t = 10 s


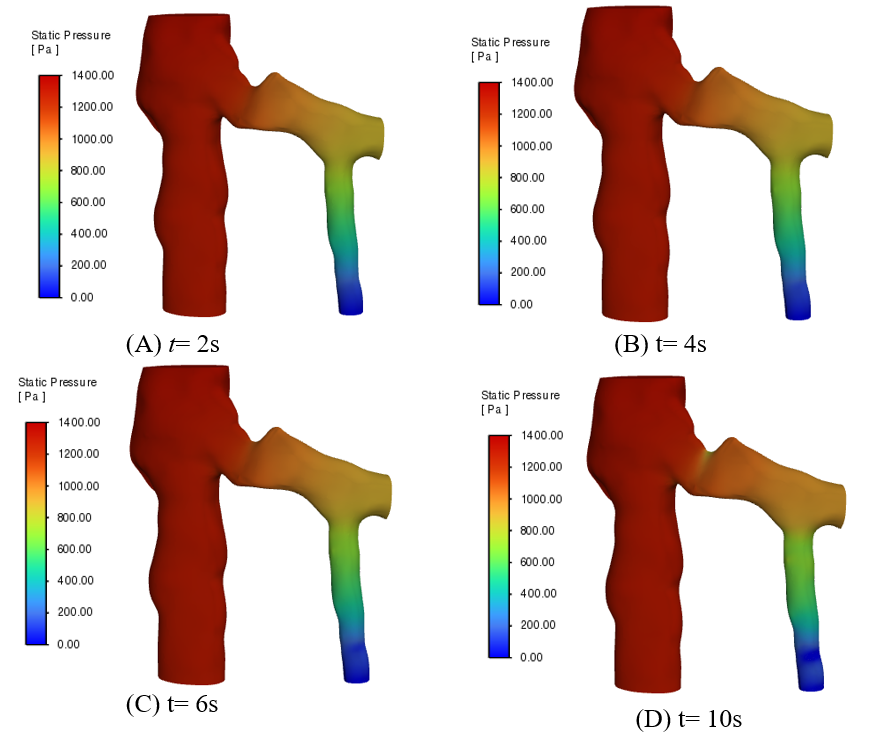


**Fig. S4**. Pressure distribution contours within the left renal vein 4·and gonadal vein at four representative time points during the Valsalva maneuver (A) Resting baseline phase, t = 2 s; (B) Ascending phase, t = 4 s; (C) Plateau phase, t = 6 s; (D) Recovery phase, t = 10 s


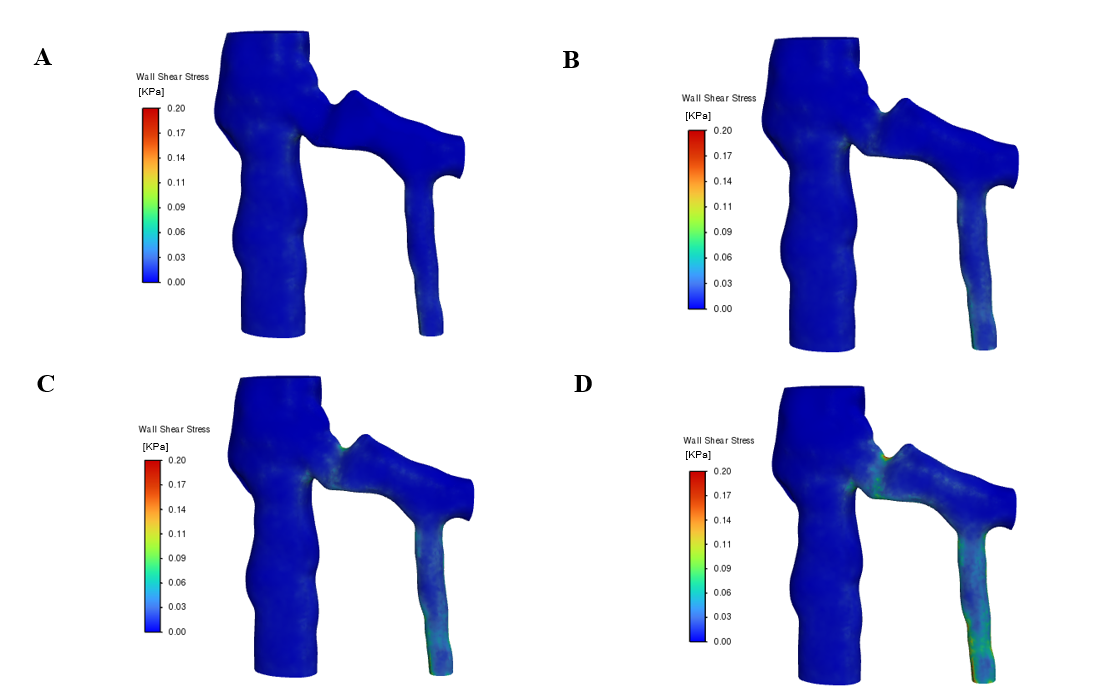


**Fig. S5**. Distribution of wall shear stress within the left renal vein and gonadal vein at four representative time points during the Valsalva maneuver (A) Resting baseline phase, t = 2 s; (B) Ascending phase, t = 4 s; (C) Plateau phase, t = 6 s; (D) Recovery phase, t = 10 s

| 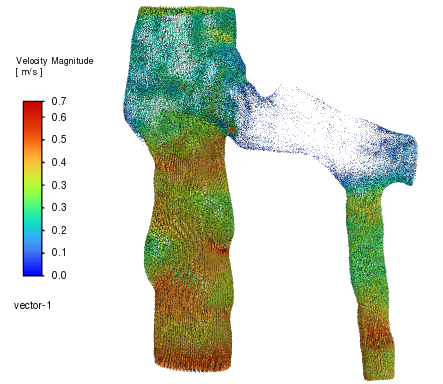 | 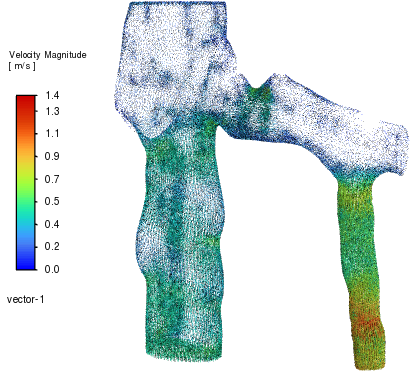 |
| --- | --- |
| (A) *t*= 2s | (B) *t*= 4s |
| 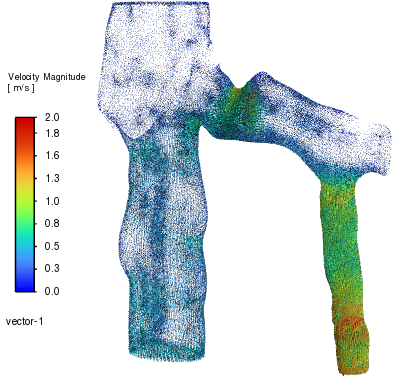 | 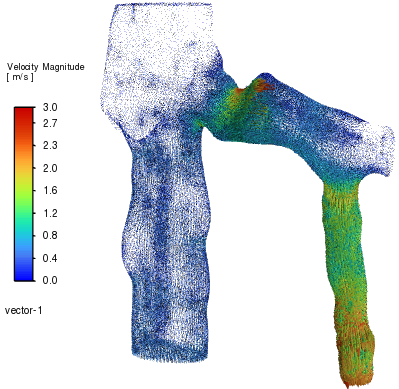 |
| (C) *t*= 6s | (D)*t*= 10s |

**Fig. S6**. Velocity and streamline distributions within the left renal vein and gonadal vein at four representative time points during the Valsalva maneuver (A) Resting baseline phase, t = 2 s; (B) Ascending phase, t = 4 s; (C) Plateau phase, t = 6 s; (D) Recovery phase, t = 10 s
